# Epidemiology, Clinical Features, Diagnosis and Management of Monkeypox Virus: A Clinical Review Article

**DOI:** 10.7759/cureus.28598

**Published:** 2022-08-30

**Authors:** Ali Ghazanfar

**Affiliations:** 1 Internal Medicine, Federal Medical and Dental College, Islamabad, PAK

**Keywords:** tecovirimat, morbidity and mortality, isolation, epidemic, epidemiology, monkey pox virus

## Abstract

The start of 2022 was marked by the sudden surge in the detection of the viral disease - monkeypox. The recent and ongoing COVID-19 epidemic makes the re-emerging of viral zoonosis particularly worrisome. The rapid spread of the monkeypox virus has sparked concerns about the start of a new epidemic. In this review, I summarize the epidemiology, clinical signs, and symptoms, transmission, diagnosis, management, and prevention of the monkeypox virus. Clinicians need to have a high index of suspicion for monkeypox in patients with high-risk factors presenting with new onset progressive rash. Patients with confirmed or suspected monkeypox infections need to be isolated until all the lesions have resolved.

## Introduction and background

**Figure 1:**
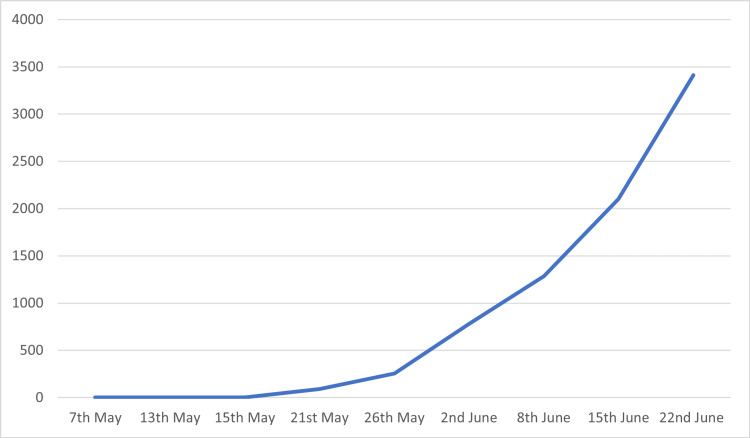
Graphical representation of monkeypox cases

The start of 2022 was marked re-emerging of the viral zoonosis; the monkeypox virus. The Monkeypox virus cases have increased from one case detected in the UK on May 7, 2022 to 1,285 cases detected in 28 countries by June 8, 2022. [1,2]. As of July 23, 2022, a total of 2,891 cases have been detected in the United States of America [3]. The rapid spread of the Monkeypox virus has sparked concerns about the start of a new epidemic. Monkeypox virus is an Orthopoxvirus from the family of poxviridae [4]. The disease has been largely limited to western and central African countries since its discovery in 1958 [4]. One of the unique things about the 2022 pandemic is that some cases had no travel history to endemic areas or history of coming in contact with anyone from endemic areas of the monkeypox virus [5]. This presents a possible unknown transmission chain that may help spread the monkeypox virus further. A meta-analysis study showed that the case fatality rate was 10.6% for the central African variant while it was 3.6% for the western African variant. The overall case fatality rate was 8.7% (7.0-10.8, 95% CI) [6]. According to a systematic review, the case fatality rate in monkeypox patients was found to vary from 1% to 11% [7]. The number of cases documented so far by World Health Organization has been presented in Table 1 and Figure 1 [1,2].

**Table 1:** Number of confirmed monkeypox cases

Date	Confirmed cases
May 7, 2022	1
May 13, 2022	2
May 15, 2022	4
May 21, 2022	92
May 26, 2022	257
June 2, 2022	780
June 8, 2022	1,285
June 15, 2022	2,103
June 22, 2022	3,413

Monkeypox shares many characteristics with the smallpox virus, which also belongs to the Orthopoxvirus family. The clinical symptoms and incubation period of monkeypox are similar to the smallpox virus [8,9]. One of the unique things about the 2022 outbreak is that most cases are not present with the classic symptoms associated with the monkeypox virus. This makes the early detection and prompt isolation of patients difficult. The monkeypox virus has been classified into two clades, western African and Congo basin (Central African). The Congo basin group is more lethal as compared to the western African clade [5].

Genetic mutations and changes have caused the pathology of the virus to change as well. A meta-analysis study concluded that in the 1970s monkeypox virus largely affected children. Due to genetic mutation, the average age of people affected by this has increased and in 2010 the average of infected individuals was found to be 21 [6]. The rapid changes that are occurring in the monkeypox virus might cause the virus to become more virulent and fatal.

## Review

Clinical signs and symptoms

Monkeypox traditionally presents with a typical picture consisting of lymphadenopathy, fever, headache, body pains, and aches. The rash affects the face and periphery more than the trunk. Mucosal membranes may also be involved including the mouth and genitals. Involvement of the corneal and conjuctival mucosa was seen in a small percentage of patients. The rash in monkeypox patients starts off as macular in appearance and then transitions into papules, vesicular, and pustules and finally drying up to form crusts that fall off [10]. The monkeypox lesions take a longer time to develop into crusts as compared to other similar diseases like chicken pox [11]. The number of lesions can also vary from a single lesion to thousands [11, 12]. Lymphadenopathy is considered as a classic feature of the monkeypox virus [13] it can be used to differentiate between monkeypox and other pox diseases. A study found that 84.2% of patients develop fever, 78.9% develop lymphadenopathy and 100% of patients develop fever and maculopapular rash [14].

A study done in 2004 found similar results. Headache and skin lesions were present in 100% of patients while 82% of patients reported fever, sweating, and chills. Coughing was reported by 73% of patients, lymphadenopathy was reported by 55% of patients and 18% patients reported malaise. Blepharitis, nausea, nasal congestion, back pain, and myalgias were reported in only 9% cases [12]. Another study concluded that nausea, vomiting, and mouth sore were independently associated with hospitalization duration of more than 48 hours [15].

Reservoir

The monkeypox virus, unlike the smallpox virus, also infects animals. Most of the animals infected appear to be monkeys or small mammals [16,17]. Several animals like monkeys, rodents, prairie dogs, and squirrels [18-20] have been identified as having the disease or containing antibodies to the monkey virus. A study done on rodents captured in Ghana showed that Graphiurus spp and cricetomys have low seroprevalence rates while funisciurus spp had a higher seroprevalence rates [21]. The funisciurus spp might be one of the natural reservoirs of the monkey virus [21]. Despite this, there is no definite information regarding the natural reservoir of the monkey virus. The task is made difficult by the dearth of virus isolates from animal species even from those that were serological positive [19]. It would appear from the numerous studies that small mammals are among the animal reservoir for the monkey virus. The international trade of exotic pets might be a possible source of the spread of the monkey virus. An outbreak in 2003 in the United States was associated with contact with infected prairie dogs. Exposure to disease animal excretions and secretions has been implicated in the spread of disease to humans [22]. The prairie dogs themselves had been infected via contact with imported rodents from Africa [22,23]. This suggests that the exotic animal trade especially that of rodents might play an important role in the spread of the monkey virus. The use of serology to detect Orthopoxvirus is problematic when trying to find an animal reservoir. Serology testing for immunoglobulin M and immunoglobulin G is nonspecific for the monkeypox virus as they are also raised with other Orthopoxvirus species [24]. There is a distinct possibility that serology tests are detecting Orthopoxvirus other than monkey virus. This complicates the investigation of finding the natural reservoir of the monkey virus as very few viruses have been isolated from the animal species.

Transmission

The route of transmission might also play an important role in the clinical symptom and spread of the virus. Reynolds et al. found that spread of the monkey virus via a bite or scratch resulted in a more severe illness with a shorter incubation period [25]. Many studies indicated that the monkey virus can spread via direct human-to-human transmission. A study done in 2015 identified sharing of drinking cups (p<0.003), food (p<0.015) and close sleeping proximity (p<0.001) were associated with the highest transmission risks [26]. Oral mucosae seem to play an important role in the spread of the virus [26]. The 2022 monkey virus spread is peculiar in regards that most of the causes are occurring among homosexuals [5]. This importance of oral mucosa and the gastrointestinal tract might explain the predominance of monkey virus among homosexuals in the 2022 outbreak. The incubation period for money pox is between six and 13 days but the range can vary from five days to 21 days [5].

Chen et al. in their study suspected that the D10L, D14L, B10R, B14R, and B19R genes might be responsible for the monkey pox virus virulence [27]. Another study done on the central African Claude showed that most of the mutations took place on the noncoding region of the genome, with the notable exception of interleukin 1 Beta gene [9]. The mutation results in a decrease of cytokine binding and decrease immune response. This mechanism might play an important role in the pathogenesis of the monkey virus as well as disease severity. Mutation of the CAR15c/18c sequences might also play a role in disease severity [9]. Studies have shown that cells infected with monkeypox virus were poorly recognized by host antiviral CD4+ and CD8+ cell [24,28]. Furthermore, monkeypox virus has been shown to infect monocytes [24]. The monkeypox virus seems to increase the production of an immunoregulator protein that suppresses immune response [24]. Recombination is a common phenomenon in poxvirus and can result in rapidly mutated virus [24]. This increases the possibility of new strains arising which may be more lethal or more infectious than the original. The central Claudia is considered more lethal than the western monkey pox Claude. Radonic et al. in a study on a scooty mangabey concluded that impaired immunity and simultaneous infection may increase monkey pox severity [29].

Diagnosis

Polymerase chain reaction (PCR) of samples taken from the patients rash is sensitive [10]. The diagnosis can be confirmed by culture, serology, and immunofluorescence assay [30]. The immunofluorescence assay and serology are less useful as there is cross-reactivity between monkeypox and other orthomyxoviruses [10,24]. The definite diagnosis of the monkeypox virus is through PCR. A study done in 2006 showed that two assays, E9L-NVAR and B6R, were 100% specific for the monkeypox virus [31]. Light Cycler quantitative PCR (LC-qPCR) has over 90% sensitivity and specificity when compared with virus isolate. The study showed that it was positive in 14 out of 15 blood samples from viral-positive monkeypox specimens [32].

Treatment

Currently, there is no approved treatment for the monkeypox virus infection. A study done in 1988 deduced that the smallpox vaccine was effective in preventing monkeypox [33]. People vaccinated with the smallpox vaccine appear to develop some degree of protection from the monkeypox virus [24]. It has been suggested that antiviral medication developed for smallpox might prove to be beneficial against the monkeypox virus. The ACAM2000^^tm^ was used in the 2003 United States monkeypox outbreak. It managed to decrease the symptoms but was ineffective in the prevention of monkeypox disease [24]. The use of IMVAMUNE in populations with high risk has been recommended by various health monitoring organizations [5,24] Tecovirimat a drug that blocks the intracellular release of the virus has shown promising results and has been recommended to be used in immunocompromised patients [5,24]. European Union (EU) body has already approved Tecovirimat for monkeypox virus while Food and Drug Administration (FDA) has approved it for smallpox. Cidofovir, ribavirin, and tiazofurin have proven to be efficacious in animal and in vitro trials [24,34,35]. Brinciofovir has been shown to have a better safety profile than Cidofovir but it has not been shown to be effective in treating Orthopoxviruses in vitro and in animal studies. A study conducted on dormouse showed that the dryvax smallpox vaccine protected against mortality by monkeypox virus [35]. More studies need to be done to establish the efficacy and safety profile of these medications.

Prevention

The current rising number of monkeypox cases might require the use of the smallpox vaccine, especially in high-risk populations. The number of people with a history of smallpox vaccination is decreasing around the world. This means that with each passing year, a greater percentage of people are at risk. World Health Organization (WHO) has approved a newer smallpox vaccine in 2019 to be used for the prevention of the monkeypox virus [10]. The number of monkeypox cases is still low despite the rising number of cases, and this presents a challenge when considering the use of the vaccine. One of the possible side effects of vaccination is the conversion of the smallpox vaccine into a pathologic strain of smallpox. One possible solution is to only implement vaccination for high-risk people and close contact with diagnosed monkeypox patients. Men who are actively engaged in sexual activities with other men and those with impaired immunity might be considered for possible vaccination. The fact that the smallpox vaccine is a live vaccine may make the use of this vaccine in severely immunocompromised patients a contraindication. Third-generation smallpox vaccine might have fewer side effects and can prove to be more beneficial in immunocompromised patients as compared to first and second generation [36].

Isolation

Patients with confirmed or suspected monkeypox infections need to be isolated until all the lesions have resolved. Home isolation is recommended for patients who have mild symptoms and who do not require hospitalization. Confirmed or suspected cases of monkeypox virus should be placed in a single-person room with a dedicated bathroom. In case the patient needs to be transported all the exposed skin lesions should be covered with a well-fitted medical mask need to be worn by the patient. Airborne isolation is needed in case of intubation. Gown, gloves, eye protection, and a respirator equipped with N95 filters or higher need to be used by the healthcare provider before entering the patient’s room [37].

Complications

The monkeypox disease can vary significantly in terms of prognosis. It mostly has a benign course yet severe complications can develop. These complications include encephalitis, nausea/vomiting leading to dehydration, and electrolyte imbalance [11,15]. Another common complication is that lesions might scar. Scarring of corneal lesions might lead to blindness [11]. Upper respiratory tract complications like tonsilitis and pharyngitis can also occur [13]. Patients that have not been vaccinated with the smallpox vaccine tend to have more severe complications as compared to vaccinated patients [13]. A study found that complications occurred in around 74% of all unvaccinated patients [13].

## Conclusions

The rapid spread of monkeypox has shown the lack of measures in place to prevent epidemics. There is a need to quarantine and check animals being internationally transported for monkeypox virus. A high index of suspicion needs to be in place when examining patients, especially the high-risk population. There is a need to initiate smallpox vaccine in high-risk populations like immunocompromised, those men engaging in homosexual acts with other men and those handling internationally transported animals, especially rodents and other small mammals. Physicians need to be aware of the early signs and symptoms of the monkeypox virus so that early diagnosis and prompt treatment can be initiated. There is a need to do more research in order to come up with more effective preventive and treatment measures.
